# Two new emerald geometrid species of *Telotheta* Warren from Ecuador and Bolivia (Lepidoptera: Geometridae, Geometrinae, Lophochoristini)

**DOI:** 10.3897/BDJ.2.e1158

**Published:** 2014-09-19

**Authors:** Aare Lindt, Jaan Viidalepp

**Affiliations:** † Estonian Museum of Natural History, Tallinn, Estonia; ‡ Estonian University of Life Sciences, Tartu, Estonia

**Keywords:** Neotropics, Ecuador, Bolivia, *
Telotheta
*, new species, *
Paromphacodes
*

## Abstract

Two new species of the lophochoristine genus *Telotheta* Warren found in Ecuador and Bolivia are described. The paper focuses on the morphological description and illustration of the wing pattern and genitalia structures of the typus generis *Telotheta muscipunctata* Dognin and the newly identified species *Telotheta unoi* and *Telotheta fresei*. The distinguishing characters of the genera *Telotheta* and *Paromphacodes* are also briefly discussed.

## Introduction

A delicate, bluish green moth from Southern Ecuador was described by Dognin 1892 as *Geometra muscipunctata*. Warren 1900 described a new genus and species also found in Ecuador (*Telotheta chlorostigma*), discussing the similarity of the species he discovered and Dognin's *Geometramuscipunctata*, highlighting the differences observed in the colour of the frons, and of the venation of the forewing.

Prout 1932, who revised the genus *Telotheta* for the first time, presented a comparative morphological characterization and synonymized the two species. Prout 1932 published a picture of this species. Pitkin (Pitkin 1996), in her analysis of Neotropical emerald moth genera, grouped *Telotheta*  with the tribe Lophochoristini. She characterized the male and female genital structures of the monotypical genus *Telotheta*. The genus-level characteristics of *Telotheta* include: hind tibia with one pair of (distal) spurs, the absence of the frenulum in females, the absence of usual transverse lines on wings, the absence of fields of modified scales on the male abdominal sternite A3. Pitkin described the female antennae as being filiform or dentate and mentioned the similarity of the genera *Oospila* Warren, 1897 and *Telotheta*, while the latter differs from the former by lacking the characteristical lophochoristine abdominal crests, which are composed of specialized, long, erect and curled scales.

Two further species of the genus *Telotheta* are described below.

## Materials and methods

The present study was initiated by attempts to identify moths found in the collections of the Estonian Museum of Natural History (EMNH, Tallinn) and in the IZBE insect collection which is deposited at the Estonian University of Life Sciences (Tartu). The material is collected by Aare Lindt.

The mounting of emerald green moths is a complicated process and the method that was used required injecting some water into the thorax, followed by keeping the moth in a container with a high air moisture environment for about 2 hours, and finally desiccating the mounted sample at around 60°C for about 12 hours. Palpi, antennae, legs and details of wing venation were measured using an ocular micrometer and binocular microscopes, using 40× magnification. The genital slides of males and females were treated using established procedures (Hardwick 1950), inspected in glycerol and embedded in Euparal and photographed ventrally. The last abdominal sternites of males were descaled by brushing, and examined, as the first genital slide did not fit with the available description of *Telotheta muscipunctata* (see the description of *Telotheta fresei*  below). Moths were mostly photographed prior to investigation of the genital structures using a Canon 300D digital camera, while genital slides were photographed with an Olympus SZ60 microscope and Leica M165C digital camera. The obtained photographs were augmented using Adobe Photoshop Elements v7 in order to improve their resolution.

## Taxon treatments

### Telotheta unoi

Lindt & Viidalepp sp. n.

urn:lsid:zoobank.org:act:DA996931-3FEE-4EF9-8DEE-E4D6FAE4AE0D

#### Materials

- **Type status:** Holotype. **Occurrence:** sex: male; **Location:** country: Ecuador; stateProvince: Napo; verbatimLocality: Carlos Julio; verbatimElevation: 950 m; verbatimLatitude: 01°15'33"S; verbatimLongitude: 77°49'27"W; decimalLatitude: -1.259167; decimalLongitude: -77.824167; **Event:** samplingProtocol: UV light sampling; eventDate: 2008-02-10; **Record Level:** institutionCode: EMNH
- **Type status:** Paratype. **Occurrence:** sex: 4 males; **Location:** country: Ecuador; stateProvince: Napo; verbatimLocality: Carlos Julio; verbatimElevation: 950 m; verbatimLatitude: 01°15'33"S; verbatimLongitude: 77°49'27"W; decimalLatitude: -1.259167; decimalLongitude: -77.824167; **Event:** samplingProtocol: UV light sampling; eventDate: 2008-02-10; **Record Level:** institutionCode: EMNH; collectionCode: IZBE

#### Description

**Figure 1.**
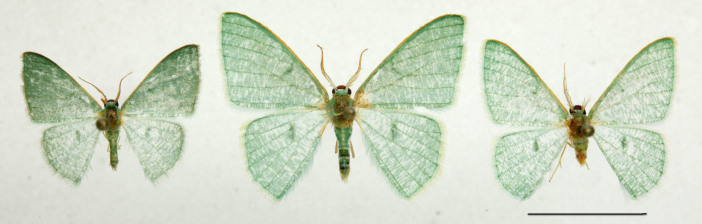
*Telotheta* moths. **Telotheta* unoi* sp. n. (left), *Telotheta fresei* sp. n. (middle), and *Telotheta muscipunctata* (right). Scale bar: 10 mm.

**Head, thorax and abdomen.** Male: The wing span of males is 15–17 mm (Fig. 1, left). The frons is almost rectangular, wide and high, dark brown above, paler towards clypeus, with a whitish line along the lower edge (Fig. 2a). The width of the frons along its dorsal edge is 0.7 mm, and along the ventral edge is 0.55 mm. The interantennal fillet is white with a green hue, and the vertex, thorax and dorsum of the abdomen are green. The male antennae are bipectinate, the pectinations being  0.5–0.55 mm long at the tenth antennal segment and tapering out beyond the middle of antenna. The venation of the wings is as described for the genus. Male palpi are slender, reaching the frons.

**Forewings** are with an angulate apex, the distal and anal margins being almost straight. The forewings are densely scaled, scales pigmented in dark green, darker towards the apex of the wings; hindwings paler, whitish green; the discal veins are more densely scaled in both wings. The discal spots are smaller than in *Telotheta fresei* and *Telotheta muscipunctata*, and dark green. The transverse lines and submarginal line is absent, the fringe being greenish (whitish in *Telotheta muscipunctata*). The costal edge of the forewing is lined light brown (slightly rosy in *Telotheta muscipunctata*). The hindwing distal margins are evenly rounded, not slightly angulated at the middle.

**Male genitalia** (Fig. 3a). The male genital armature is similar to the armatures of *Telotheta fresei* and *Telotheta muscipunctata* but smaller: the height of the genital capsule, from the tip of the uncus to the tip of the vinculum is 1.0 mm. The valva is apically bilobed, the lobes are more slender in *Telotheta unoi*  compared to those in related species. The dorsal margin of the dorsal lobe bears a row of stout curved setae in *Telotheta muscipunctata* and *Telotheta fresei*, which are lacking in the new species *Telotheta unoi*. The valva subapical lobe is edged straight ventrally, the strong setae are longer towards the base of the lobe. The anellar complex is broadest in the middle, the dorsal tips of the juxta converging. The distal projections of the sternite A8 are slender, rigid and finely rugose at the lateral edges in *Telotheta unoi*  and *Telotheta muscipunctata*, while in *Telotheta fresei*  they appear projecting flat and triangularly. The aedeagus is slightly curved, 1.0 mm long, without cornuti.

#### Etymology

The new species is dedicated to Mag. Uno Roosileht from the Estonian Museum of Natural History, coleopterist, colleague and co-traveller. The gender of the newly described species is masculine.

#### Distribution

The new species was collected in the middle elevation tropical mountain forests on the Eastern slope of the Eastern Cordilleras, in Ecuador.

#### Taxon discussion

Remarks. Forewings of the new species *Telotheta unoi*  are dark green, hindwings greenish white. This wing pattern — forewings darker than hindwings — is similar to that of an another Neotropical genus, *Paromphacodes* Warren. However, *Paromphacodes* is different in the venation of wings (Prout 1932) and in the presence of two pairs of spurs on the hind tibia. Moreover, the species of *Paromphacodes* studied have rosy or red-brown postmedial spots on the forewings and the fringe of wings usually contrastingly red-brown or rosy (Pitkin 1996, Prout 1932). *Paromphacodes* has nemoriine male genitalia, very different from those in *Telotheta*.

### Telotheta muscipunctata

(Dognin, 1892)

#### Materials

- **Type status:** Other material. **Occurrence:** recordedBy: Aare Lindt; individualCount: 1; sex: male; **Location:** country: Ecuador; stateProvince: Pichincha; verbatimLocality: El Transito; verbatimElevation: 1080; verbatimLatitude: 00°18'59"S; verbatimLongitude: 78°54'54"W; **Event:** samplingProtocol: UV light sampling; eventDate: 2008-02-03; **Record Level:** institutionCode: ENHM

#### Description

Both original descriptions by P. Dognin and W. Warren (see Introduction) refer to the pale bluish green moths with large green discal blotches on both wings. The colour of the frons was described as yellowish ochreous by Dognin, and as brick red by Warren.

Prout 1912, Prout 1932 synonymized the two names and Pitkin 1996 redescribed the genus and presented illustrations of the genital structures, including the bicornute last abdominal sternite found in males.

Additional data: The wing span is 16 mm long in our specimen (Fig. 1, right). The frons is slender, trapezoidal, higher than wide (Fig. 2b), with a thin, whitish line below. The male antenna with pectinations up to 0.8 mm lomg. Wings are covered with semi-transparent white and green scales, white scales prevailing while green scales giving moths a "punctate" appearance. Male genital armatures of the three species are very similar one to another. *Telotheta muscipunctata* has a row of about 20 stout curved setae to the distal margin of valva, and the thorn-like projections of the sternite A8 appear slightly diverging (Fig. 3b).

#### Distribution

The species is attributed a wide distribution range in the research literature, from Costa Rica and Venezuela to Peru in Western South America (Pitkin 1996). However, the primary types of this species, as well as our material, are from the Western Cordilleras in Ecuador. See the description of *Telotheta fresei* sp. n.

### Telotheta fresei

Lindt & Viidalepp sp. n.

urn:lsid:zoobank.org:act:C474D8D5-F21E-4537-A58B-D602AD603466

#### Materials

- **Type status:** Holotype. **Occurrence:** sex: male; **Location:** country: Ecuador; stateProvince: Morona Santiago; verbatimLocality: Plan de Milagro; verbatimElevation: 2080; verbatimLatitude: 03°00'25"S; verbatimLongitude: 78°30'11"W; **Event:** samplingProtocol: UV light sampling; eventDate: 2007-04-27; **Record Level:** institutionCode: EMNH
- **Type status:** Paratype. **Occurrence:** sex: male; **Location:** country: Ecuador; stateProvince: Morona Santiago; verbatimLocality: Plan de Milagro; verbatimElevation: 2080 m; verbatimLatitude: 03°00'25"S; verbatimLongitude: 78°30'11"W; **Event:** samplingProtocol: UV light sampling; eventDate: 2007-04-24; **Record Level:** collectionCode: IZBE
- **Type status:** Paratype. **Occurrence:** sex: 2 males; **Location:** country: Ecuador; stateProvince: Morona Santiago; verbatimLocality: Gualaquiza; verbatimElevation: 1570 m; verbatimLatitude: 03°17'58"S; verbatimLongitude: 78°33'59"W; **Event:** samplingProtocol: UV light sampling; eventDate: 2007-04-23; **Record Level:** collectionCode: IZBE
- **Type status:** Paratype. **Occurrence:** sex: 1 male; **Location:** country: Ecuador; stateProvince: Pastaza; verbatimLocality: Cuasha; verbatimElevation: 880 m; verbatimLatitude: 01°50'01"S; verbatimLongitude: 77°39'01"W; **Event:** samplingProtocol: UV light sampling; eventDate: 2007-04-28; **Record Level:** institutionCode: EMNH
- **Type status:** Paratype. **Occurrence:** sex: 2 males, 2 females; **Location:** country: Ecuador; stateProvince: Napo; verbatimLocality: Cotundo; verbatimElevation: 1080 m; verbatimLatitude: 00°41'28"S; verbatimLongitude: 77°43'56"W; **Event:** samplingProtocol: UV light sampling; eventDate: 2007-05-19; **Record Level:** institutionCode: EMNH; collectionCode: IZBE
- **Type status:** Paratype. **Occurrence:** sex: 2 males, 2 females; **Location:** country: Ecuador; stateProvince: Napo; verbatimLocality: Sarayacu; verbatimElevation: 1900 m; verbatimLatitude: 00°38'51"S; verbatimLongitude: 77°49'00"W; **Event:** samplingProtocol: UV light sampling; eventDate: 2007-05-20; **Record Level:** institutionCode: EMNH; collectionCode: IZBE
- **Type status:** Paratype. **Occurrence:** sex: 1 male; **Location:** country: Ecuador; stateProvince: Zamora Chinchipe; verbatimLocality: Zamora; verbatimElevation: 1000 m; verbatimLatitude: 04°06'30"S; verbatimLongitude: 78°57'49"W; **Event:** samplingProtocol: UV light sampling; eventDate: 2007-04-19; **Record Level:** institutionCode: E;NH
- **Type status:** Paratype. **Occurrence:** sex: 2 males, 2 females; **Location:** country: Ecuador; stateProvince: Zamora Chinchipe; verbatimLocality: Los Encuentros; verbatimElevation: 1460; verbatimLatitude: 03°54'40"S; verbatimLongitude: 78°36'39"W; **Event:** eventDate: 2007-04-21; **Record Level:** institutionCode: EMNH; collectionCode: IZBE
- **Type status:** Paratype. **Occurrence:** sex: 2 males; **Location:** country: Bolivia; stateProvince: Cochabamba; verbatimLocality: Naranjitos; verbatimElevation: 630 m; verbatimLatitude: 17°03'32"S; verbatimLongitude: 65°38'44"W; **Event:** samplingProtocol: UV light sampling; eventDate: 2010-10-04; **Record Level:** institutionCode: EMNH
- **Type status:** Paratype. **Occurrence:** sex: 2 females; **Location:** country: Bolivia; stateProvince: Cochabamba; verbatimLocality: ., N. P. Carrasco; verbatimElevation: 900 m; verbatimLatitude: 17°06'44"S; verbatimLongitude: 65°33'55"W; **Event:** samplingProtocol: UV light sampling; eventDate: 2010-10-05; **Record Level:** institutionCode: EMNH
- **Type status:** Paratype. **Occurrence:** sex: 1 male; **Location:** country: Bolivia; stateProvince: Caranavi prov.; verbatimLocality: Caranavi; verbatimElevation: 1200 m; verbatimLatitude: 15°43'19"S; verbatimLongitude: 67°29'07"W; **Event:** samplingProtocol: UV light sampling; eventDate: 2010-11-05; **Record Level:** collectionCode: IZBE
- **Type status:** Paratype. **Occurrence:** sex: 1 male; **Location:** country: Bolivia; stateProvince: Inicua; verbatimLocality: Quiquibei; verbatimElevation: 550 m; verbatimLatitude: 15°30'16"S; verbatimLongitude: 67°11'52"W; **Event:** samplingProtocol: UV light sampling; eventDate: 2010-10-31; **Record Level:** collectionCode: IZBE
- **Type status:** Paratype. **Occurrence:** sex: 1 male; **Location:** country: Bolivia; stateProvince: La Paz; verbatimLocality: Sacramento Alto; verbatimElevation: 2000 m; verbatimLatitude: 16°14'44"S; verbatimLongitude: 67°47'09"W; **Event:** samplingProtocol: UV light sampling; eventDate: 2010-11-08; **Record Level:** collectionCode: IZBE

#### Description

**Head, thorax and abdomen.** Male: Wing span 18–22 mm. Female: 20–25 mm (Fig. 1, right). The frons is of almost rectangular shape (Fig. 2c), slightly bulging and broader than in other species of this genus, being brown with a white line below. The width of frons along its dorsal edge is 0.9–1.0 mm, along the ventral edge 0.7–0.8 mm. The male antennae are bipectinate, the pectinations being up to 0.70–0.75 mm long at the tenth segment, tapering out beyond the middle of the antenna. The female antennae are finely serrated. The haustellum is present, the palpi have a thin 3rd segment, hardly projecting in front of the frons in males, longer in females. The interantennal fillet is white with a green hue, the vertex and thorax are green, the dorsum of the abdomen is green, mottled with white. The venation and structure of the wings as described for the genus (Pitkin 1996), the hindwing veins Rs and M_1 _are on a very long stalk and the distal margin of the hindwing is slightly angulate at the end of the vein M_3_. The discal veins are straight in both wings. The male hind tibia is slender, without the proximal pair of spurs and without a hair pencil, the distal spurs are long. The facies of the moths is similar to that in *Telotheta muscipunctata*.

**Forewings** (Fig. 1) are densely scaled with semi-transparent white and green scales as in *Telotheta muscipunctata*, the transparent scales are mostly pigmented in green to one quarter of their apical part. The hindwings are concolorous, both wings having a large green discal spot. The transverse lines and submarginal line are absent, the fringe is whitish as in *Telotheta muscipunctata* (greenish in *Telotheta unoi*). The forewing costal edge is lined light brown (slightly rosy in *Telotheta muscipunctata*).

**Male genitalia** (Fig. 3c). The male genital armature of *Telotheta fresei* is similar to that of *Telotheta unoi* and *Telotheta muscipunctata* but large (the height of the genital capsule is 1.0 mm for *Telotheta unoi*, 1.3 mm for *Telotheta fresei* and for *Telotheta muscipunctata*). The aedeagus is about 1.3 mm long. The distal process of the uncus is reduced, the gnathos arms are short hooked. In *Telotheta fresei* the juxta and the transtilla together constitute a long anellar complex. The juxta is broader than in the two other species. The valva is apically bilobed, the apical lobe as in *Telotheta muscipunctata* (more slender in *Telotheta unoi*). The dorsal margin of the apical lobe bears about 8-10 scattered, stout, curved setae, and about 20 such setae in a row in *Telotheta muscipunctata*. The ventral margin of the subapical lobe of the valva is straight. Sternite A8 distal projections are flat and triangularly rounded, completely different from the slender, rigid projections in *Telotheta muscipunctata* and *Telotheta unoi*.

The female genitalia (Fig. 4a)  are composed of a short ductus bursae, a pyriform bursa copulatrix and the signum is a small plate of trapezoidal shape, without any lateral prongs (Fig. 4b). The roundish sclerotization to the ostial region is much weaker than in *Telotheta muscipunctata* (compare Fig. 223 in Pitkin 1996). The anterior apophyses are much shorter than the posterior apophyses.

**Variation.** Both males and females of this new species were collected together from three collecting sites in Ecuador (Cotundo, Sarayacu and Los Encuentros) and therefore treated as conspecific. Pitkin 1996 has examined both primary types of the known species. She characterized *Telotheta muscipunctata* males by the presence of two rod-like processes to the posterior edge of the last abdominal sternite and females by having a large roundish sclerotization in  the sterigmal area, a membranous bursa copulatrix, provided with a "small signum with two tapered prongs" (Pitkin 1996: 408). There was no need to re-examine the types, as the morphological differences between *Telotheta muscipunctata* and *Telotheta fresei* are clear.

The series of *Telotheta fresei* from Ecuador and from Bolivia differ slightly in the shape of the eighth sternite of male abdomen. The sternite has triangular projections to its posterior edge in the populations of Ecuador, and more rounded projections in the  populations from Bolivia. This phenomenon requires further investigation: the differences are slight and, possibly, clinal.

#### Etymology

This new species is named in honour of the former lutherian pastor in Lääne-Nigula, West Estionia, Theodor Alexander Benedict Frese. His insect collection, consisting of several thousands of mounted and labelled local butterflies, moths and other hexapods, was donated in 1864 to the Eestimaa Provintsiaalmuuseum, the precedor of the Estonian Museum of Natural History. The 150th anniversary of the Museum will be celebrated 2014. The gender is masculine.

#### Distribution

*Telotheta fresei* is described from Eastern Ecuador and Bolivia.

#### Ecology

*Telotheta fresei* is collected in tropical forests between about 500 m and 2000 m elevation.

## Identification Keys

### Key to *Telotheta* species

**Table:** 

1	Forewing and hindwing concolorous (Fig. 1).	2
–	Forewing dark green, hindwing light green (Fig. 1)	*Telotheta unoi* sp. n.
2	The last abdominal sternite of male with two thorn-like projections (Fig. 3b). The signum bicornute in female.	*Telotheta muscipunctata*
–	The last abdominal sternite of male flat with two triangular processes (Fig. 3c).	*Telotheta fresei* sp. n.

## Discussion

The three *Telotheta* species are to be separated as follows:

*Telotheta muscipunctata*  moths have a slender frons, pale green, semidiaphanous wings and a bicornute sternite A8 in male;

*Telotheta fresei* moths have frons broad, both wings similarly pale green, the male sternite A8 flat with two broad projections distally;

*Telotheta unoi*  has frons broad, forewings dark green and dense scaled, and the male sternite A8 bicornute like in *Telotheta muscipunctata*.

## Supplementary Material

XML Treatment for Telotheta unoi

XML Treatment for Telotheta muscipunctata

XML Treatment for Telotheta fresei

## Figures and Tables

**Figure 2a.**
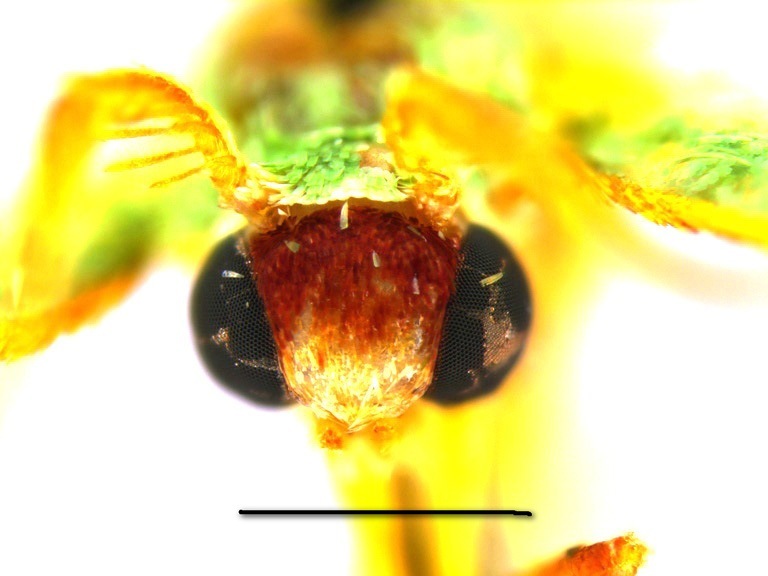
*Telotheta unoi* sp. n.

**Figure 2b.**
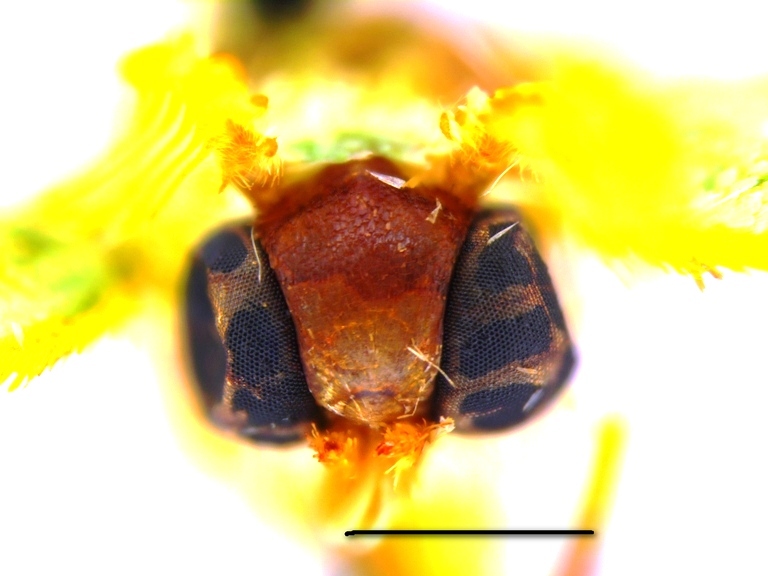
*Telotheta muscipunctata*

**Figure 2c.**
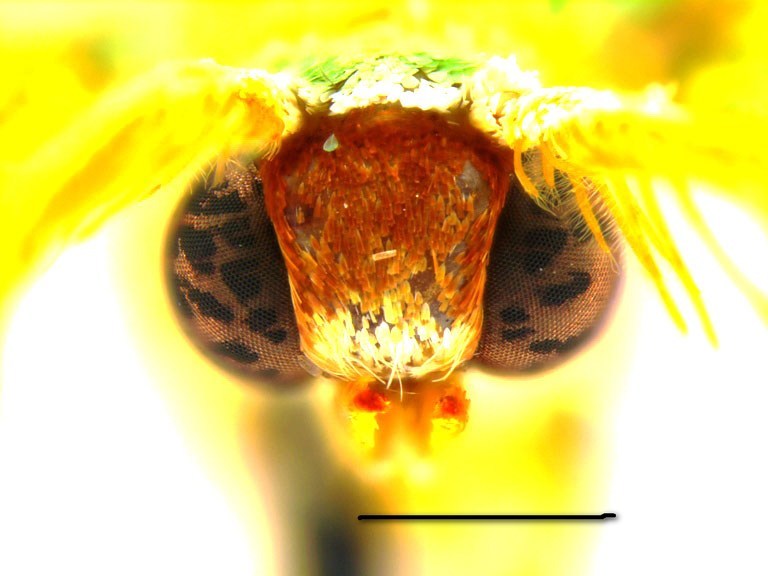
*Telotheta fresei* sp. n.

**Figure 3a.**
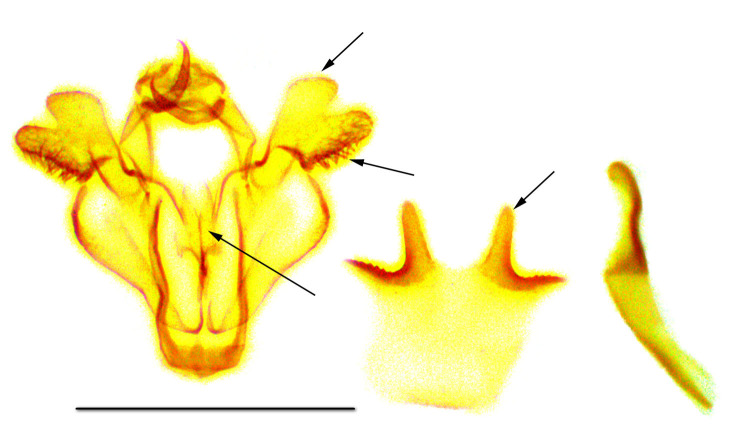
*Telotheta unoi* sp. n.

**Figure 3b.**
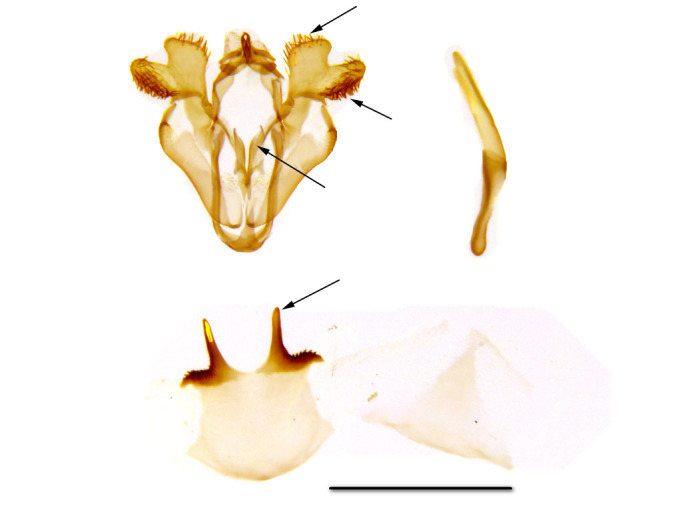
*Telotheta muscipunctata*

**Figure 3c.**
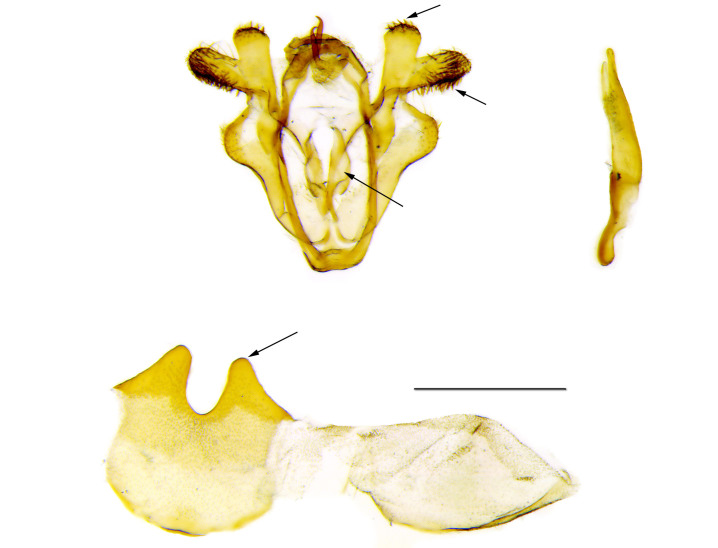
*Telotheta fresei* sp. n.

**Figure 4a.**
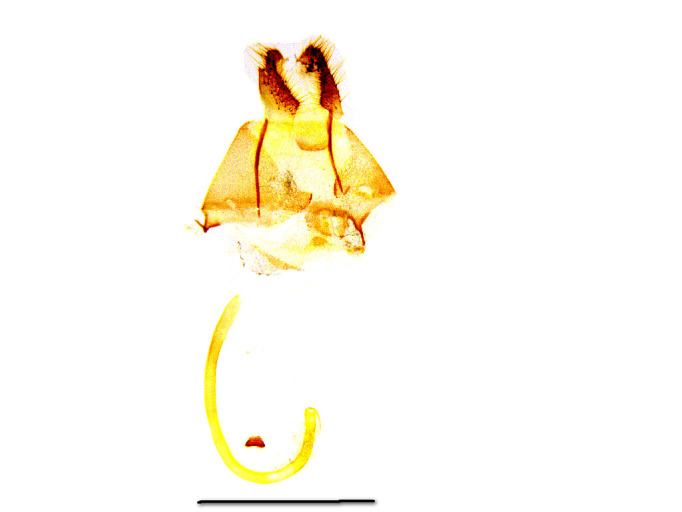
female genital armature. Scale bar: 1 mm

**Figure 4b.**
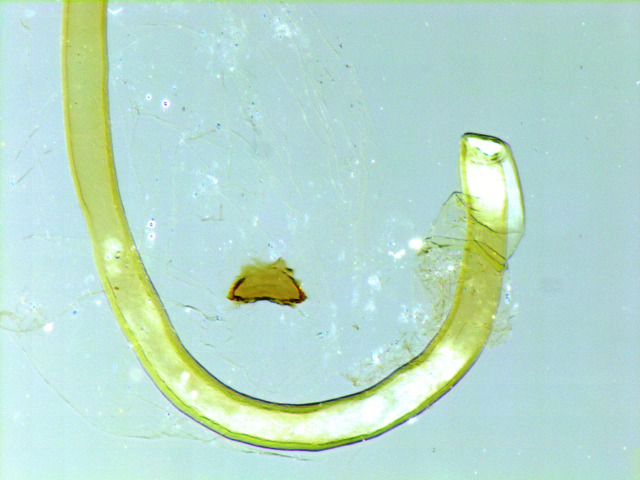
signum, enlarged
